# Use of supercritical carbon dioxide technology for fabricating a tissue engineering scaffold for anterior cruciate ligament repair

**DOI:** 10.1038/s41598-020-70994-z

**Published:** 2020-08-20

**Authors:** Ines Sherifi, Manon Bachy, Thomas Laumonier, Hervé Petite, Didier Hannouche

**Affiliations:** 1 Laboratoire de Bioingénierie et Biomécanique Ostéo-Articulaire (B2OA), UMR, CNRS 7052, Paris 7 University, Paris, France; 2 Division of Cardiology, Department of Medicine, Weill Cornell Medical College, New York Presbyterian Hospital, New York, NY USA; 3 Department of Pediatric Orthopaedic Surgery, AP-HP, Hôpital Trousseau, Paris, France; 4 Department of Orthopaedic Surgery, Faculty of Medicine, Geneva University Hospitals, Avenue Gabrielle Perret Gentil 4, 1205 Geneva, Switzerland

**Keywords:** Biotechnology, Biomaterials, Biomedical materials

## Abstract

Tissue-engineered grafts may be useful in Anterior Cruciate Ligament (ACL) repair and provide a novel, alternative treatment to clinical complications of rupture, harvest site morbidity and biocompatibility associated with autografts, allografts and synthetic grafts. We successfully used supercritical carbon dioxide (Sc-CO_2_) technology for manufacturing a “smart” biomaterial scaffold, which retains the native protein conformation and tensile strength of the natural ACL but is decellularized for a decreased immunogenic response. We designed and fabricated a new scaffold exhibiting (1) high tensile strength and biomechanical properties comparable to those of the native tissue, (2) thermodynamically-stable extra-cellular matrix (ECM), (3) preserved collagen composition and crosslinking, (4) a decellularized material milieu with potential for future engineering applications and (5) proven feasibility and biocompatibility in an animal model of ligament reconstruction. Because of the “smart” material ECM, this scaffold may have the potential for providing a niche and for directing stem cell growth, differentiations and function pertinent to new tissue formation. Sc-CO_2_-related technology is advanced and has the capability to provide scaffolds of high strength and durability, which sustain a lifetime of wear and tear under mechanical loading in vivo.

## Introduction

The anterior cruciate ligament (ACL) is the most frequently injured ligament in the knee. More than 200,000 patients are diagnosed yearly with ACL disruptions^1,2^ and approximately 120,000 ACL reconstructions are performed annually in the United States^3^. Because it receives nourishment mainly from the surrounding synovial fluid, the ACL has poor natural healing ability and thus necessitates surgical reconstruction when ruptured^4^. Primary surgical repair of ligaments has shown poor results in clinical practice and is not commonly used as a treatment option today^5^. Since the reconstructed knees need to last the lifetime of the patient, the repaired ligament needs to possess reliable durability under the body’s mechanical loading and active stress during daily activities. This requirement translates into the need for a biomechanically and biologically stable replacement that must withstand a life-time of wear and tear.

Historically, the medical field has explored various approaches in search of the perfect tissue substitute for the natural ACL. Autografts, which involve reconstruction using the patient’s own tendons to replace the damaged ACL, have offered good strength and served as successful surgical substitutes in 85–90% of clinical cases^6^. However, autograft harvesting causes significant donor site morbidity, including anterior knee pain, patellar tendinitis, and infra-patellar contracture, following the harvesting of the patellar tendon; hamstring weakness, and saphenous nerve injury after harvesting of the hamstring tendons^7^. Synthetic materials have been extensively studied as replacements of the native ACL^8,9^. These synthetic substitutes have generally exhibited long-term propensity for undesirable stretching or rupture, and inability to withstand the wear and tear of the knee joint resulting from long-term use under mechanical loading^2^. In addition, synthetic materials have exhibited various types and extent of incorporation into surrounding tissue at the implantation site, various grades of insufficient tissue maturation post-implantation and, sometimes, have caused knee joint inflammation as a result of the innate immune response to foreign bodies^10^.

These clinical needs and challenges have prompted biomedical researchers to consider tissue engineering approaches in order to produce a biocompatible ACL implant exhibiting long-term durability in vivo^1,11^. Current tissue engineering strategies maintain that the signals needed by cells for tissue regeneration-related functions are not provided by the synthetic biomaterials designed and produced to date^12^. The differences between synthetic biomaterials and materials produced by nature over millions of years are staggering. For example, the instructive extracellular matrix (ECM) of collagenous tissues exhibits high molecular diversity in its supramolecular structure including, but not limited, to 14 different collagens, 18 isoforms of laminin, 7 additional proteins including elastin and glycoproteins, 12 known proteoglycans, as well as a great array of growth and morphogenesis factors^12,13^. In addition, the “perfect” biomaterial for implant prostheses needs to: (1) be biodegradable and non-toxic in its complete form and its degradation products; (2) have similar biomechanical properties as the replaced native tissue; (3) promote cell functions pertinent to new tissue formation including attachment, migration, production of ECM, etc.; (4) be a substrate for bioactive chemical compounds (such as enzymes, medications, growth factors); (5) exhibit high angiogenic potential with low immunogenicity and low thrombogenicity; (6) be malleable for processing into various sizes, geometric forms and structures; and (7) ideally possess high information content like the ECM^14^.

In the present study, we designed, formulated and evaluated the efficacy of a “smart” scaffold for ACL tissue engineering applications using supercritical carbon dioxide (Sc-CO_2_) technology. This strategy employs naturally-occurring allograft ACL material and uses the advantages of the Sc-CO_2_ method in order to obtain a biocompatible scaffold for ACL tissue engineering applications. Sc-CO_2_ technology was proposed for biomedical engineering applications due, in part, to the fact that CO_2_ is non-flammable, non-toxic, physiologically biocompatible, chemically inert and readily available in nature^15–17^. When CO_2_ is heated and compressed above its critical point (7.38 MPa and 304.2 K, respectively), it exhibits the density of a liquid but the diffusivity and viscosity of a gas^18^. These properties allow CO_2_ to penetrate porous structures as well as prevent pore collapse due to the lack of a vapor–liquid interface and minimized surface tension, which is particularly important in physiological applications that require the use of non-toxic solvent alternatives^19^. In addition, compressed CO_2_ can also be used in biomaterial sterilization processes because it has the ability to kill bacteria, without altering the composition and the properties of the materials^20^. Sc-CO_2_ technology has been proposed for the sterilization of highly-sensitive materials, such as medical devices, polymeric biomaterials, and grafts^21^.

To the best of our knowledge, they are only few reports of the use of Sc-CO_2_ technology to produce scaffolds for tissue engineering applications^22–24^. In the present study, using Sc-CO_2_ strategy, we designed and developed a new ligament scaffold with preserved mechanical properties, collagen content and cross-linking, and which could have the potential for providing a niche for stem cell expansion and function pertinent to new tissue formation.

## Methods

### Harvest of native tissue

Rabbit hindlegs were surgically dissected and the Extensor Digitorium Longus (EDL), the Flexor Digitorium Fibularis (FDF), and the Calcaneous Communis (commonly referred to as the Achilles (A)) tendons were harvested. The sizes of these tendons in the rabbit hindleg are approximately 2.3 cm, 8.8 cm, and 3.4 cm for the EDL, FDF, and A tendons, respectively.

### Supercritical carbon dioxide treatment

A Separex Equipments® Supercritical Extraction Unit (Nancy, France) was used for the treatment of the excised tendon specimens. The machine was run at a flow rate of 5 kg/h and contained a 4 L extraction vessel. During this procedure the tendons were each placed within open sterile glass containers and were hermetically sealed within pouches composed of two different materials surfaces: (1) an impenetrable Tyvek® 2FS and (2) a porous polyethyleneterephthalate/polyethelene (PET/PE). This packaging unit allowed visualization and identification of the treated samples, maintained sterility of the specimens within the bags and glass containers, and allowed easy diffusion of the supercritical fluid through the highly porous surface of the pouches. In order to determine the effect of supercritical carbon dioxide on the structure and mechanical strength of the tissues, two supercritical process parameters were tested: *cond1* (pressure = 102.3 ± 3.0 bars; temperature = 40.6 ± 0.7 °C; values are mean ± S.D.) and *cond2* (pressure = 147.0 ± 5.7 bars; temperature = 44.3 ± 0.7 °C; values are mean ± S.D.). Rabbit legs were randomly assigned to three groups (*cond1, cond2,* and control). The limbs were weighed, radiographed for femur and tibia length measurements (Table 1), and the tendons were then dissected and treated alone in the Separex Sc-CO_2_ machine. The pressure within the Separex machine was increased gradually within 20–30 min to the *cond1* and *cond2* parameters. The target temperature and pressure were sustained for either 1 or 2 h. At the end of each cycle, the extraction vessel was gradually depressurized within 20–30 min. The samples were preserved within the hermetically-sealed pouches and containers at 4 °C until further testing.

**Table 1:** Supercritical carbon dioxide cycles and samples descriptive characteristics.

Group	Pressure (bar)	Temperature (°C)	Supercritical CO_2_ treatment (hours)	Leg weights (g)*	Femur lengths (cm)*	Tibia lengths (cm)*
Condition 1	100 bar	40 °C	1	239 ± 30	8.37 ± 0.24	8.95 ± 0.57
Condition 2	150 bar	45 °C	2	219 ± 13	8.11 ± 0.45	8.76 ± 0.20
Control	Atmospheric pressure	RT	None	226 ± 15	8.33 ± 0.49	9.16 ± 0.30

*There is no statistical difference between femur lengths and control (*p* = 0.16 and *p* = 0.83 respectively for *cond1* and *cond2*). There is no statistical difference between the tibia lengths and control (*p* = 0.40 and *p* = 0.35 respectively for *cond1* and *cond2*). There is no statistical difference between the leg weights in the two groups and control (*p* = 0.43 and *p* = 0.41 respectively for *cond1* and *cond2*).

### ***Mechanical testing of native and Sc-CO***_***2***_***-treated tendons***

Two sandpaper pieces (number 80, size: 2.0 × 1.8 cm^2^) were affixed (using Super Glue number 3) to the respective ends of each tendon ensuring that an equal and uniform segment of each tendon specimen was contained between the sheets. An Instron® 4,505 electromechanical testing machine (Instron Corp., Canton, MA, USA) was used to test the performance of the tendons under traction. Force versus distance curves and graphs were obtained for each tendon tested.

### Analysis of mechanical properties

The tendon cross-sectional area was approximated as an ellipse and the following formula was used to calculate its surface area, S^25^:

1 $$ S = \pi ab $$

where *a* and *b* refer to the elliptical radii. The displacement of each specimen under tension was measured in millimetres (mm) using the Instron® machine. Force versus displacement graphs provided information regarding the performance of the tendons under traction and were used to calculate the stiffness, *E,* (N/ mm) of the tendons with the following equation^26^:

2 $$ Stiffness = \Delta F{/}\Delta l $$

where ∆F refers to the change in applied force and ∆l refers to the change in length of the tested specimen. Stiffness is an extensive material property and a measure of the resistance offered by an elastic body to deformation such as bending, stretching and compression. The force–displacement curves were used to calculate the nominal stress, *σ*_*nominal*_, using the following formula^27^:

3 $$ \sigma_{nominal} = F{/}S_{o} $$

where *S*_*o*_ is the initial surface area before each specimen was placed under traction. Given that the volume of the material remained constant during traction, the Cauchy stress tensor, *σ*_*Cauchy*_, was calculated as follows^28^:

4 $$ \sigma_{Cauchy} = F(l_{o} + \Delta l){/}S_{o} l_{o} $$

where F refers to the measured force (N), *l*_*o*_ to the initial length (mm) of the specimen tested, *∆l* to the change in the specimen length (mm) under stress, and *S*_*o*_ (mm^2^) to the initial calculated specimen surface area using Eq. 1. In addition, the strain of the material, ε, was calculated as follows^26^:

5 $$ \varepsilon = \Delta l{/}l_{o} $$

6 $$\upvarepsilon _{integrated} = \ln (1 +\upvarepsilon ) $$

where ε refers to the strain on the specimen tested. The stress and strain data were then used to calculate the Young’s Modulus, (E; in MPa) for the native and Sc-CO_2_-treated specimens.

### Histological analysis

The native (n = 5) and Sc-CO_2_-treated tendons (n = 5) were immersed into 4% formaldehyde (pH 7.38) within 48 h of dissection and treatment in the Separex Equipments® Supercritical Extraction Unit. Each tissue specimen was sectioned and processed for histology using established techniques. Histology sections were examined using a DMRXA Leica microscope (Leica Microsystèmes SAS, Rueil-Malmaison Cedex, France).

### Quantification of tendon collagen content

The following reagents were purchased from Sigma-Aldrich (St-Louis, MO, USA): cis-4-Hydroxy-l-proline, 4-dimethylaminobenzaldehyde, chloramine-T hydrate, perchloric acid, sodium acetate trihydrate, and glacial acetic acid. Citric acid and propanolol were purchased from Prolabo (Lyon, France). Sodium hydroxide was purchased from Fluka Biochemica (Switzerland). All aforementioned chemicals were of analytical grade. The acetate-citrate buffer was prepared using 120 mg of sodium acetate trihydrate, 46 g of citric acid, 12 mL of glacial acetic acid, and 34 g of sodium hydroxide; this solution was brought to 1 L using distilled water and the pH was adjusted to 6.5. The chloramine-T reagent solution was prepared by dissolving 1.27 g of chloramine-T hydrate in 20 mL of 50% propanolol; this solution was brought to 100 mL using acetate-citrate buffer. Ehrlich’s reagent was prepared freshly before each assay by dissolving 15 g of *p*-dimethylaminobenzaldehyde in 2:1 propanolol/perchloric acid solution (v/v). For the standard curve preparation, stock of hydroxyproline (at an initial concentration of 1 mg/mL) was used to make successive dilutions in the range from 2 to 10 µg/mL. Aliquots of 100 µL of these dilutions were oxidized using 900 µL of chloramine T solution at room temperature for 25 min and then mixed gently with 1 mL of Ehrlich’s aldehyde reagent (for the development of the chromophore). The optical density of each triplicate sample was determined using a spectrophotometer at 550 nm. The average values of these results were plotted as the optical density versus hydroxyproline concentration (µg/mL).

### Calorimetry analysis

The FDF control group was frozen at − 60 °C immediately after dissection whereas the experimental group of tendons was first treated in supercritical carbon dioxide and then frozen at − 60 °C. All samples were allowed to thaw slowly at room temperature (RT) and were analyzed using a DSC823e Mettler-Toledo Calorimeter (Mettler Toledo International Inc, Zurich, Swittzeland). The calorimetry equipment capsules used for the analysis were 40 µL in volume. A 1–2 mm long segment of each sample of interest to the present study was cut and placed in the capsules for testing. The heat flow cycles in the calorimeter were run from 20 to 100 °C at a temperature increase rate of 2 °C/min. The weighted enthalpy (*∆H*_*scaffold*_) corresponding to the energy absorbed by the tissue during helix-coil transformation, the temperature of denaturation (*T*_*denaturation*_) at maximum heat absorption, and the mid-peak width (*W*) were determined from the curve using a software integrated with the calorimeter.

### Analysis of collagen crosslinking

Native and Sc-CO_2_-treated EDL tendons were dissected, minced into 1–2 mm pieces, suspended in 1 mL per 10 mg tissue of potassium phosphate buffer (at pH 7.6), and stirred continuously for 72 h. These samples were then reduced using 1% w/v of sodium tetrahydridoborate (NaBH4) solution in 0.01 M sodium hydroxide (NaOH). The reaction was allowed to proceed at 37 °C for 1 h and it was stopped by adding acetic acid. The samples were washed four times with distilled water, centrifuged, and were lyophilized overnight. The reduced materials were then hydrolyzed with 6 N hydrochloric acid at 110 °C for 24 h in sealed glass tubes and dried *in vacuo*. These specimens were dissolved in sodium citrate buffer (pH 2.20), filtered through a 0.45 µm pore diameter filter and analyzed using High Performance Liquid Chromatography (Biorad, Muchen, Germany). Enzymatically immature (dihydroxylysinonorleucine DHLNL, hydroxylysinonorleucine HLNL) and mature (pyridinoline PYD, deoxypyridinoline DPD) collagen crosslinks were measured using high performance liquid chromatography-Electrospray Ionization Mass Spectrometry (HPLC-ESI-MS) according to a previously published method^29^. To determine the level of collagen crosslinking, a portion of the NaBH_4_-reduced and lyophilised tendon was hydrolysed using hydrochloric acid 6.0 mol/L (HCl 6 N) and pre-treated on Solid-phase Extraction (SPE) columns crosslinks Chromabond (Macherey Nagel GmbH & Co.KG, Düren, Germany) to remove interfering fluorophores. DHLNL, HLNL, PYD and DPD were separated on an Alliance 2,695 separation module (Waters Corp., Milford, MA, USA) using an Atlantis® T3, 3 µm, 4.6 × 100 mm reversed phase column protected by an Atlantis® T3, 3 µm 4.6 × 20 mm guard cartridge (Waters Corp., Milford, MA, USA) and were quantified using a Waters Micromass® ZQ™ Single Quadrupole Mass Spectrometer (Waters Corp., Milford, MA, USA). Separation of the crosslinks was achieved using a gradient solution. Solvent A consisted of 0.12% HBFA, and solvent B was 50% acetonitrile. The separation column was equilibrated using 10% solvent B prior to use for the collagen analysis. The flow rate was 1.0 mL/min and the column temperature 25 °C. Separation of the crosslinks took place within the first 40 min of Solvent B gradient from 10 to 20%. The electrospray ionization (ESI) source was operated in the positive ion mode. The target ions were [M + H]+ at m/z 308 for DHLNL, 292 m/z for HLNL, [M]+ m/z 429 for PYD, and m/z 413 for DPD.

### ***Culture of native and Sc-CO***_***2***_***-treated tendon explants and fibroblasts***

In order to evaluate the presence of viable cells within the native (n = 5) and Sc-CO_2_-treated tendons (n = 5), tendon explant cultures were prepared from the specimens, according to a previously described procedure^30^. Strips of native and Sc-CO_2_-treated tendons were rinsed with PBS, transferred to 6-well plates, and cultured in Dulbecco’s modified Eagle’s medium (DMEM) supplemented with 10% (v/v) fetal bovine serum (FBS; Hyclone), 1 M HEPES buffer solution (Life Technologies), 100× nonessential aminoacid mixture (Life Technologies), 100× l-glutamine (Life Technologies), 100 U/mL penicillin/streptomycin (Life Technologies), 100× sodium pyruvate (Life Technologies). Tendon explants were maintained in culture for 10 days in a 5% CO_2_ incubator at 37 °C, with a medium change every 3 days.

In a second experiment, cells were extracted from native (n = 5) and Sc-CO_2_-treated tendons (n = 5), and cultured in vitro. Each tendon sample was washed twice with PBS and minced into 1–2 mm pieces. They were then treated with collagenase at 37 °C with vortexing every 20 s for 2 h, using established procedures^31^. The reaction was stopped using 3 mL of α- MEM medium supplemented with 10% BSA at room temperature. Tissue debris were removed using a 100-µm strainer. Cells were counted and plated at an initial density of 2 × 10^6^ nucleated cells per cm^2^ and cultured in a 5% CO_2_ incubator at 37 °C in the same culture medium. Culture medium was replaced every 3 days thereafter until cells reached 70% confluence.

### In vivo experiment

As a proof-of-concept study, the treated tendon was also implanted in a rabbit model of ACL using a procedure previously described^32^. The protocol was approved by the Animal Experiment Ethics Committee of Lariboisiere-Villemin Number 09 (CEEA-LV/2010-01-01). All experiments were performed in accordance with the guidelines of the French Agriculture and Forestry Ministry for handling animals (decree 87,849, license A75-05-22). We implanted two skeletally mature female New Zealand white rabbits (age, 26 weeks; weight, 3.5–4 kg). Two EDL tendons were harvested for one New Zealand white rabbit, treated with Sc-CO_2_, and prepared as a replacement construct for implantation. The graft was implanted in the right knee, the other knee served as the control. Briefly, the knee joint capsule was opened surgically in three rabbits and, after excision of the ACL remnants, tibial and femoral tunnels were created and the graft was implanted isometrically to the native ACL position. Finally, the graft was sutured in situ using 3.0 polypropelene sutures to the periosteum. Animals were allowed to move freely after the operation. Animals were sacrificed at 12 days, and the joints were collected for macroscopical evaluation.

### Statistical analysis

Numerical data were reported as mean ± SD. Parameter errors were determined using propagation of error analysis. Comparisons were made by one-factor analysis of variance (ANOVA). A *p* value of < 0.05 was considered indicative of significance.

## Results

### ***Mechanical properties of native and Sc-CO***_***2***_***-treated tendons***

**Figure 1:**
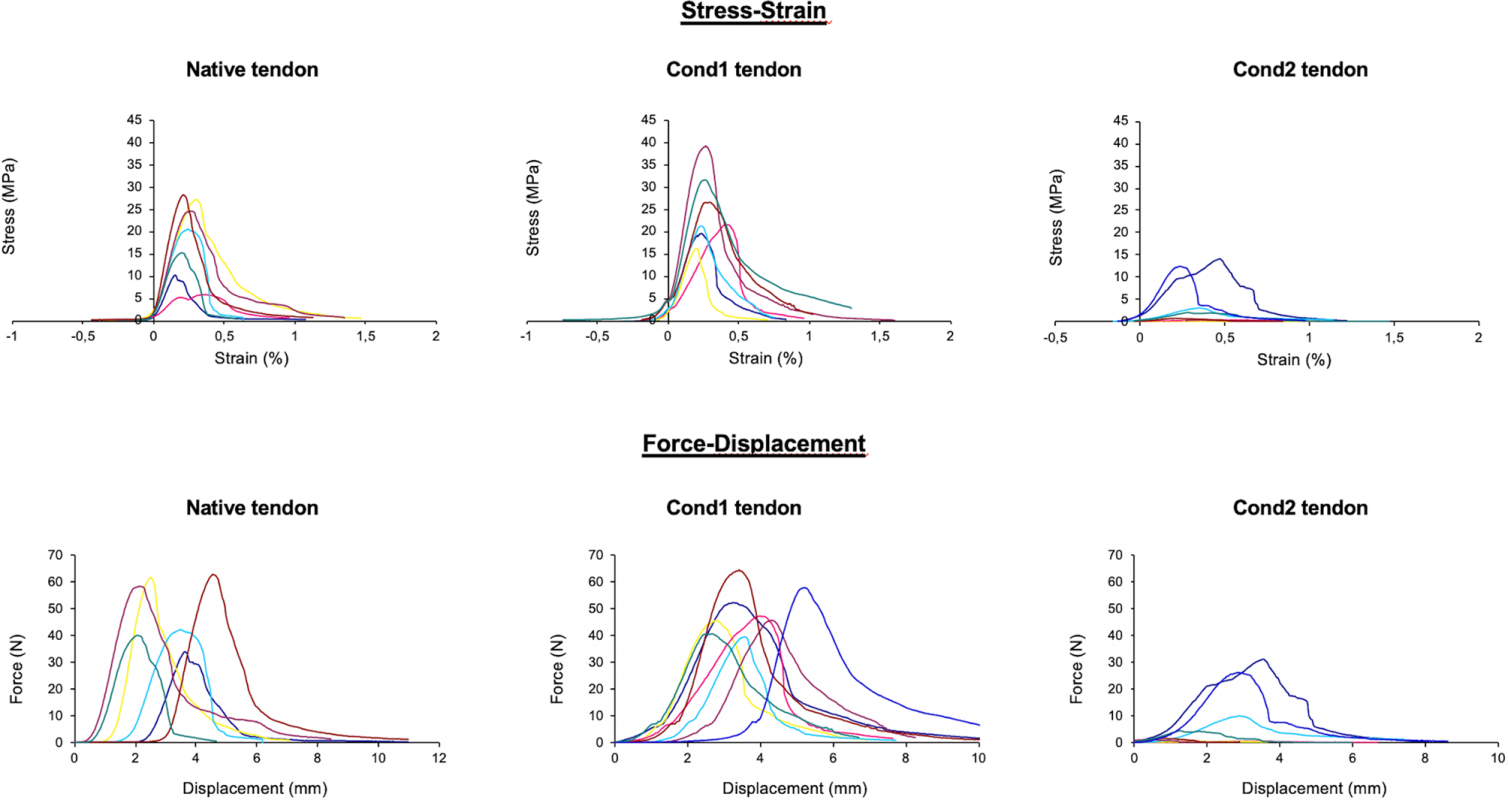
Force–displacement and stress–strain relationships for the scaffolds prepared using *cond1* and *cond2* were determined and compared with the respective parameters of the native tendons. The color lines indicate the results observed for 7 samples.

Force–displacement and stress–strain relationships for the scaffolds prepared using *cond1* and *cond2* were determined and compared with the respective parameters of the native (*control*) tissue (Fig. 1). Biomechanical parameters for the native non-treated tendon were biomaterial stiffness (*S*_*scaffold*_), Nominal Young’s Modulus (*E*_*nominal*_) and Cauchy Young’s Modulus (*E*_*Cauchy*_) of 40 ± 18 N/mm, 105 ± 43 MPa, and 121 ± 48 MPa, respectively. A single-factor multigroup analysis of variance revealed that the results in all three groups (n = 7 scaffolds) tested were significantly (*p* < 0.05) different. The scaffold produced with *cond1* exhibited similar biomechanical properties with the control native tissue. In contrast, the respective parameters of scaffolds formulated via *cond2* exhibited markedly decreased (*p* < 0.05) biomechanical properties (Table 2).

**Table 2:** Biomechanical results.

Group	Biomaterial stiffness (N/mm)	Nominal Young’s modulus (MPa)	Cauchy Young’s modulus (MPa)
Condition 1	35.15 ± 12.01	116.82 ± 40.28	146.59 ± 49.97
Condition 2	9.30 ± 6.89*	18.96 ± 24.44*	24.29 ± 30.32*
Control	40.10 ± 17.94	104.52 ± 42.76	120.59 ± 48.25

*There is a statistical difference between *cond2* and control in all biomechanical parameters including biomaterial stiffness, nominal young’s modulus and the Cauchy young’s modulus with *p* < 0.05. No statistical difference in any of the categories between *cond1* group and control group. Single factor ANOVA undertaken for the three groups for all the categories shows the groups to be statistically different with *p* < 0.005.

### Histological analysis

We analysed tendons from native tissues and only from engineered scaffold using *cond1* based on the results of the mechanical evaluation.

**Figure 2:**
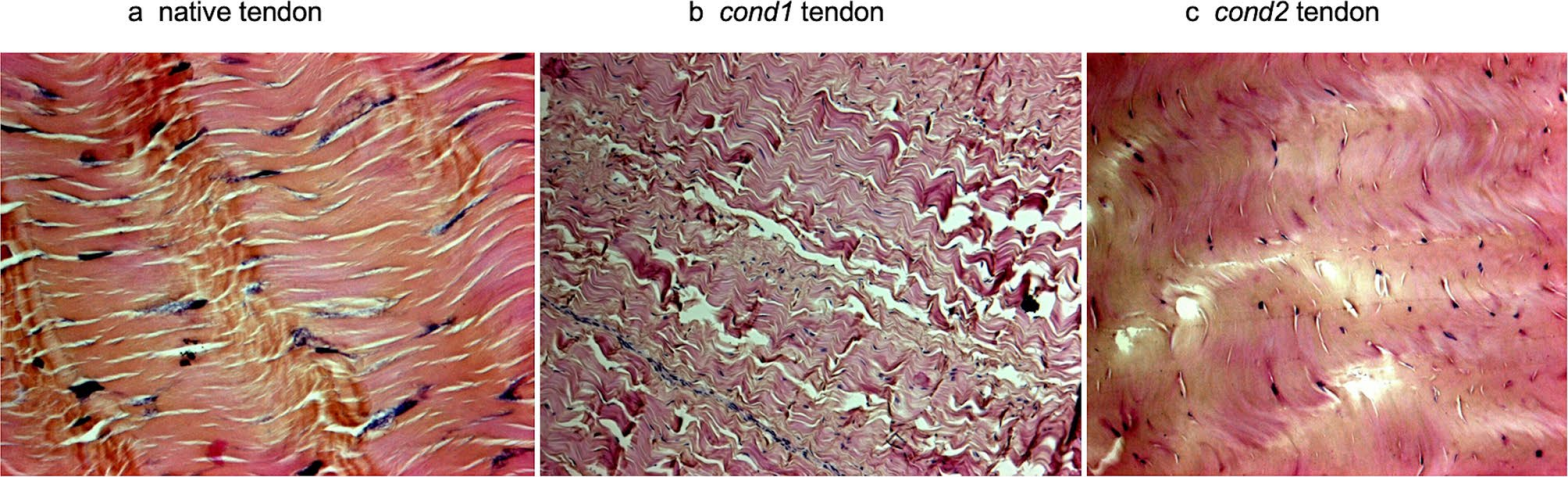
Histologcial analysis (H&E staining) of native tendon (**a**), tendon treated with *cond1* (**b**) and tendon treated with *cond2* (**c**). Images shown are representative of 5 independent experiments (20× magnification).

Comparison of the histology results obtained from the native tendon with those from *cond1* scaffolds revealed the following: (1) increased waviness of the specimens; (2) increased number of spaces within the collagen fibrils; and (3) changed morphology of the cell nuclei (from elongated to smaller rounded nuclei). Histology differences between the native tissue and *cond2* scaffolds were as follows: (1) disappearance of distinct collagen fibril lines as the tissue assumed a “merged” configuration; (2) change of the cell nuclei shapes (from elongated to rounded ones); and (3) increased number of spaces and incidences of tears between the fibrils (Fig. 2).

### Quantification of tendon collagen content

In order to better characterize the biochemical composition of the new treated scaffold, the collagen composition of the matrices was determined via hydroxyproline quantification. Sc-CO_2_-treated tendons contained the same amount of collagen than the native tendons composition (*p* = 0.26). The treated and the native tendons contained 75.5 ± 7.5% and 76.4 ± 10.7% of collagen, respectively.

### Calorimetry analysis

**Figure 3:**
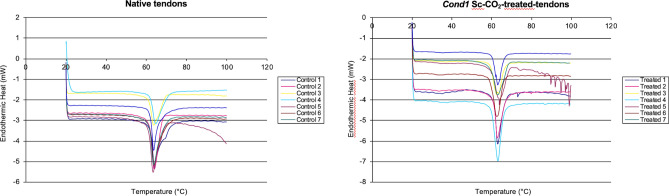
Graphical representation calorimetry analysis of Sc-CO_2_-treated tendons under *cond1* and the native tendons. *∆H*_*scaffold*_ and *T*_*denaturation*_ values for the *cond1* tendons were significantly (*p* < 0.04 and *p* < 0.0012, respectively) different than the respective controls.

Calorimetric analysis was used to compare the weighted enthalpy (*∆H*_*scaffold*_), temperature of denaturation (*T*_*denaturation*_), and mid-peak width (*W*) of native tissue and tendons prepared using *cond1* (n = 8). This analysis yielded values of 12.45 ± 2.19 J/g, 62.96 ± 0.45 °C and 0.70 ± 0.12 cm for *∆H*_*scaffold*_, *T*_*denaturation*_ and *W,* respectively, for Sc-CO_2_-treated tendons using *cond1*, and values of 14.87 ± 2.11 J/g, 63.79 ± 0.35 °C and 0.69 ± 0.10 cm, respectively, for control tendons. *∆H*_*scaffold*_ and *T*_*denaturation*_ values for the *cond1* tendons were significantly (*p* < 0.04 and *p* < 0.0012, respectively) different than the respective controls (Fig. 3).

### Analysis of collagen crosslinking

Crosslinks were measured in mmol/mol of collagen and used to compare the scaffold treated with Sc-CO_2_-treated tendons under *cond1* and the native tendons: dehydroxyleucinenorleucine (DHLNL), hydroxyleucinenorleucine (HLNL), pyridinium (PYD) and deoxypyridinium (DPD).

**Figure 4:**
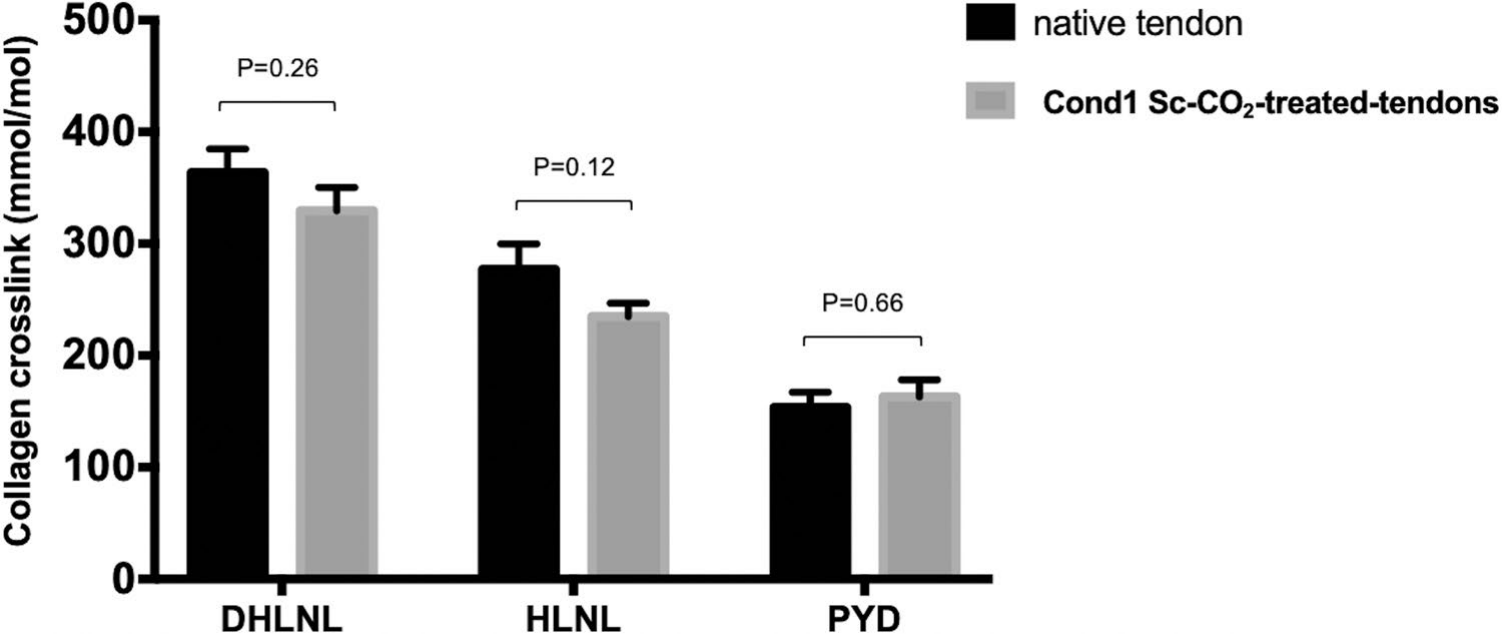
Graphical representation collagen crosslinking of Sc-CO_2_-treated tendons under *cond1* and the native tendons. Enzymatically immature (dihydroxylysinonorleucine DHLNL, hydroxylysinonorleucine HLNL) and mature (pyridinoline PYD) collagen crosslinks were measured. An ANOVA statistical analysis revealed that these values were not statistically significant from each other with respective p values of 0.26, 0.12, 0.66 and 0.11.

The graphical representation of this comparison is depicted below (Fig. 4) with the DHLNL, HLNL, PYD and DPD values being 364 ± 59 mmol/mol collagen, 277 ± 64 mmol/mol collagen, 154 ± 39 mmol/mol collagen and 1.27 ± 0.71 mmol/mol collagen respectively for the native tendons. The respective values for the treated tendons (*cond1*) were 330 ± 59 mmol/mol collagen, 235 ± 33 mmol/mol collagen, 163 ± 43 mmol/mol collagen and 0.83 ± 0.16 mmol/mol collagen. These values (n = 8 for both groups) were not statistically significant from each other with respective p values of 0.26, 0.12, 0.66 and 0.11.

### ***Culture of native and Sc-CO***_***2***_***-treated tendon explants and fibroblasts***

**Figure 5:**
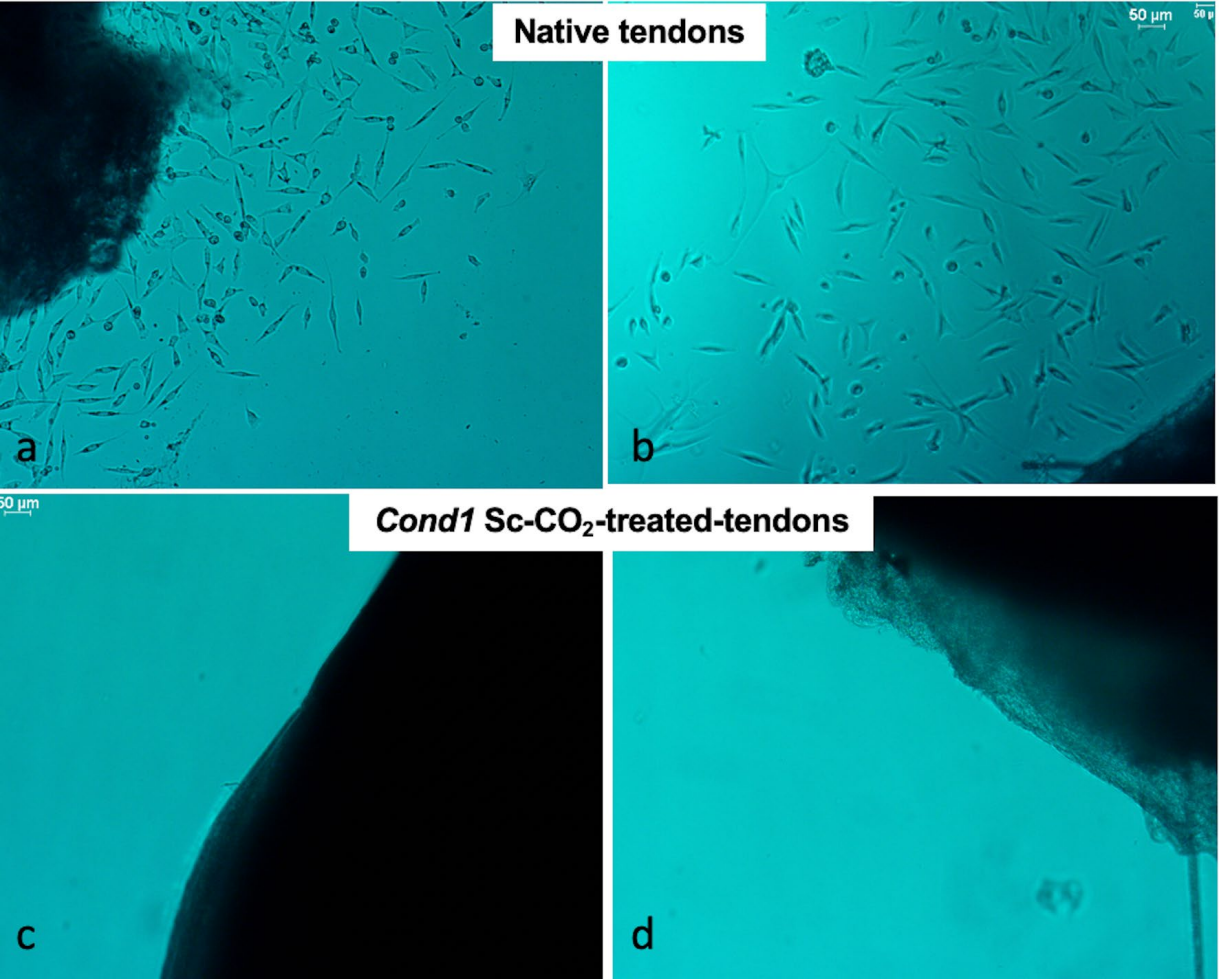
Cell Growth Assay (50 μm scale). (**a**,**b**) Untreated Tendons, cells observed at 7 days in two control samples. (**c**,**d**) Treated Tendons (*cond1*): no cells growth is observed in either sample. Images shown are representative of 5 independent experiments (scale bar: 50 μm).

Viability of fibroblasts was assessed using cells from cultured tissue explants and the fibroblast extraction assay (Fig. 5A,B). No cells from the treated tissue extracts survived. In contrast, fibroblasts from the native control tendons (which had not been treated with supercritical carbon dioxide) survived and proliferated for many weeks (Fig. 5C,D). Cells were not characterized, as it was not the purpose of the present study.

### In vivo experiment

The in vivo experiment was a proof of concept to determine if the implantation of treated Sc-CO_2_-tendons was feasible, and to evaluate the gross appearance and early behaviour of the grafts. Given the biomechanical properties of Sc-CO_2_-treated tendons using *cond1*, they could be handled and manipulated in the surgery room without any preparatory procedure. The grafts could be grasped with surgical instruments, passed within the femoral and tibial tunnels, and sutured without damaging them. Twelve days after implantation, dissection revealed a continuous graft, which was firm to palpation and glistening within the flexed joint. There were no signs of inflammation, the joint fluid was clear, and the synovium appearing healthy, somewhat thickened.

## Discussion

Tendon allografts for ACL repair are gaining popularity worldwide, as their safety and efficacy continue to improve^33^. However, increased risks of revision have been reported with the use of allografts, irrespective of whether the grafts were processed with chemicals or with low- or high-dose irradiation^34,35^. In the present study, a “smart” porous scaffold, with preserved biomechanical properties, was designed and fabricated using supercritical carbon dioxide technology (Sc-CO_2_). Excised rabbit tendons were used as the “natural” materials and were hermetically sealed in containers composed of one impenetrable Tyvek® 2FS surface and one porous polyethyleneterephthalate/polyethelene (PET/PE) surface. In this environment, diffusion of Sc-CO_2_ was enhanced by the use of highly porous packaging, which also maximized sterility and specimen separation.

Since biological tissues are subjected to various mechanical stresses (such as strain, shear and pressure), one main objective of the tissue engineering design was to ascertain the biomechanical profile of the tissue constructs. In order to determine the effect of Sc-CO_2_ on the structure and mechanical strength of highly tensile tissues such as ligaments and tendons, two supercritical parameters were tested. The collected data were compared to the biomechanical profile of the native tissue of the same species. Sc-CO_2_-treated tendons under *cond1* exhibited similar biomechanical properties than the native tendons. Since collagen crosslinking increases the mechanical stability and strength of materials^36^, our scaffolds fabricated using *cond1* had preserved collagen crosslinking, which provides supporting evidence for the efficacy of the material. We observed a slightly higher cross-linking content for PYD in Sc-CO_2_-treated tendons (163 ± 43 mmol/mol collagen vs. 154 ± 39 mmol/mol collagen), although this was not statistically significant. A possible explanation of the higher PYD cross-links and lower DHLNL and HLNL cross-links in the treated tendons could be that Sc-CO_2_ may convert the intermediate immature cross-links derived from the hydroxylysine pathway into more stable mature pyridinoline cross-links. However, this could not be verified, as the content of DPD, another trivalent mature cross-link, was slightly lower in the treated tendons (0.83 ± 0.16 mmol/mol collagen) as compared to the controls (1.27 ± 0.71 mmol/mol collagen) (*p* = 0.11). It is more likely that the differences observed are simply due to the natural variation in our data set.

Electron-beam and gamma irradiation are commonly used methods of treating tendon allografts for ACL repair, although they severely damage the ECM at irradiation doses much lower than those required for sterilization of tissue grafts prior to implantation^33,37–39^. In a recent paper, Bui et al.^40^ compared the biomechanical properties of allograft menisci treated with either Sc-CO_2_ or a 25 kGy gamma irradiation. Although both treatments yielded an increase in stiffness and stress relaxation as compared to control group, samples treated with Sc-CO_2_ had significantly less alteration of their biomechanical properties. Another group^41^ evaluated the stiffness, failure stress, and failure load of tendon allografts sterilized with either Sc-CO_2_ or 2.0–2.8 Mrad gamma irradiation. Sc-CO_2_ method resulted in significantly lower stiffness than unprocessed and irradiated grafts, which is not consistent with our results. This might be explained by the significantly smaller size of tendons used in the Sc-CO_2_ group in their study, and by the supercritical process parameters (temperature and pressure) used that could have been different. Moreover, the authors stated that the samples were placed in a chamber with CO_2_ that was pressurized and heated to the point where CO_2_ forms a solvent; however, the exact process parameters were not provided in their manuscript. We also showed that Sc-CO_2_-treated tendons under two different conditions exhibited significantly different biomechanical properties, providing evidence that the Sc-CO_2_ process can be optimized to produce tissue engineering scaffolds of highly tensile strength. Nichols et al.^42^ also compared the efficacy of Sc-CO_2_ and 15–25 kGy gamma radiation in achieving the sterilization of musculoskeletal allografts, including bone-to-bone patellar tendons. Sterilization runs were conducted using a peracetic acid-based additive charged with CO_2_ at a pressure of 99 bars and a temperature of 35 ± 3 °C for 6–9 mn. They could achieve a sterility assurance level of 10^−6^, while preserving the biomechanical properties of the tendons, as compared to untreated samples. In contrast, gamma irradiation significantly impacted the biomechanical properties in terms of maximal load and overall toughness. Similar results were reported by Sun et al.^43^ in a recent study. The authors treated 10-mm long ovine tendons with Sc-CO_2_ combined with two different reagents, one containing peracetic acid and hydrogen peroxide (NovaKill™), and one containing sodium dodecyl sulfate (SDS), a well-known decellularizing detergent. They showed that ultimate tensile stress and Young’s modulus of tendons treated with Sc-CO_2_, NovaKill™, and 0.1% SDS were significantly higher compared to 25 kGy-irradiated tendons and SDS treated tendons. Ultrastructural morphology of Sc-CO_2_ treated tendons was considered intact, when examined by scanning electron microscopy (SEM) and Fourier transform infrared (FTIR) spectroscopy. Notably, the authors did not evaluate the effect of Sc-CO_2_ treatment alone, neither they tested different parameters, such as pressure, temperature, treatment time, which are known to be of paramount importance for the efficacy of the supercritical treatment^21^. Also, they acknowledged that tendon decellularization could not be achieved with their protocols despite the use of two different additives. Biological scaffolds composed of decellularized extracellular matrix have recently gained increased attention in regenerative medicine strategies, in order to reduce the host tissue response related to remnants of cellular material^44^. Decellularization is usually achieved using chemical reagents and enzymes, which are powerful and efficient tools in eliminating cell and genetic material from tissues. However, they often have negative effects, in terms of structural, biochemical, and biomechanical properties, which are crucial to support the function, the repopulation, and the growth of the newly formed tissue^45,46^. In this endeavour, Sc-CO_2_ has been successfully proposed by several authors to decellularize bone^24,47^ and soft tissue allografts^41^, including heart valves^48^ and heart myocardium^23^. In our study, Sc-CO_2_-treated tendons could be decellularized devoid of viable cells and cell remnants. In contrast, fibroblasts from the native control tendons survived and proliferated for several weeks in vitro. Cells were not characterized, as this was not the purpose of the present study.

Differential scanning calorimetry (DSC) was used to determine the thermal stability and protein unfolding interactions in the matrix of the scaffold prepared using *cond1* (Table 3; Fig. 3). DSC analysis is a powerful technique used in cell biology to determine thermodynamic properties of biomacromolecules as well as the thermodynamic stability of proteins, polynucleotides, and lipid assemblies present in ECM^49^. Although *cond1* scaffolds exhibited mechanical properties similar to those of the native control tendon tissue, we were able to deduce a 0.83 °C (1.3%) difference in the thermal transition temperature, and a reduction of 2.42 J/g (3.8%) in denaturation enthalpy, which indicates a very mild reduction in collagen thermal stability. Giannini et al.^50^ examined the effect of fresh-freezing at − 80 °C on human posterior tibial tendon, which is a common procedure used for preservation of human tendon allografts. DSC showed a 5.4% decrease in mean denaturation temperature, and 14% decrease in denaturation enthalpy. Reports of calorimetry studies provided evidence that gamma-irradiation of tissues at 12 kGy yielded an even greater denaturation of cross-linked collagen fibers, with an average decrease in *T*_*denaturation*_ of 22.8 °C (38%) when not-hydrated and 17.9 °C (30%) when hydrated^51^. These changes are significantly higher than those obtained in our study, supporting our hypothesis that Sc-CO_2_ is protective with respect to the thermodynamic properties of the tested tendons.

**Table 3:** Calorimetry data.

Sample	Weighted enthalpy (J/g)	Denaturation peak (°C)	Mid-peak width (cm)
Condition 1	12.45 ± 2.19*1	62.96 ± 0.45*2	0.70 ± 0.12*3
Control	14.87 ± 2.11*1	63.79 ± 0.35*2	0.69 ± 0.10*3

*There is a statistical difference between *cond1* and control in the Weighted Enthalpy of the material and the denaturation peak with *p* < 0.05 (*1: *p* = 0.04; *2: *p* = 0.0012). There is no statistical difference between the mid-peak widths (*3: *p* = 0.82).

**Figure 6:**
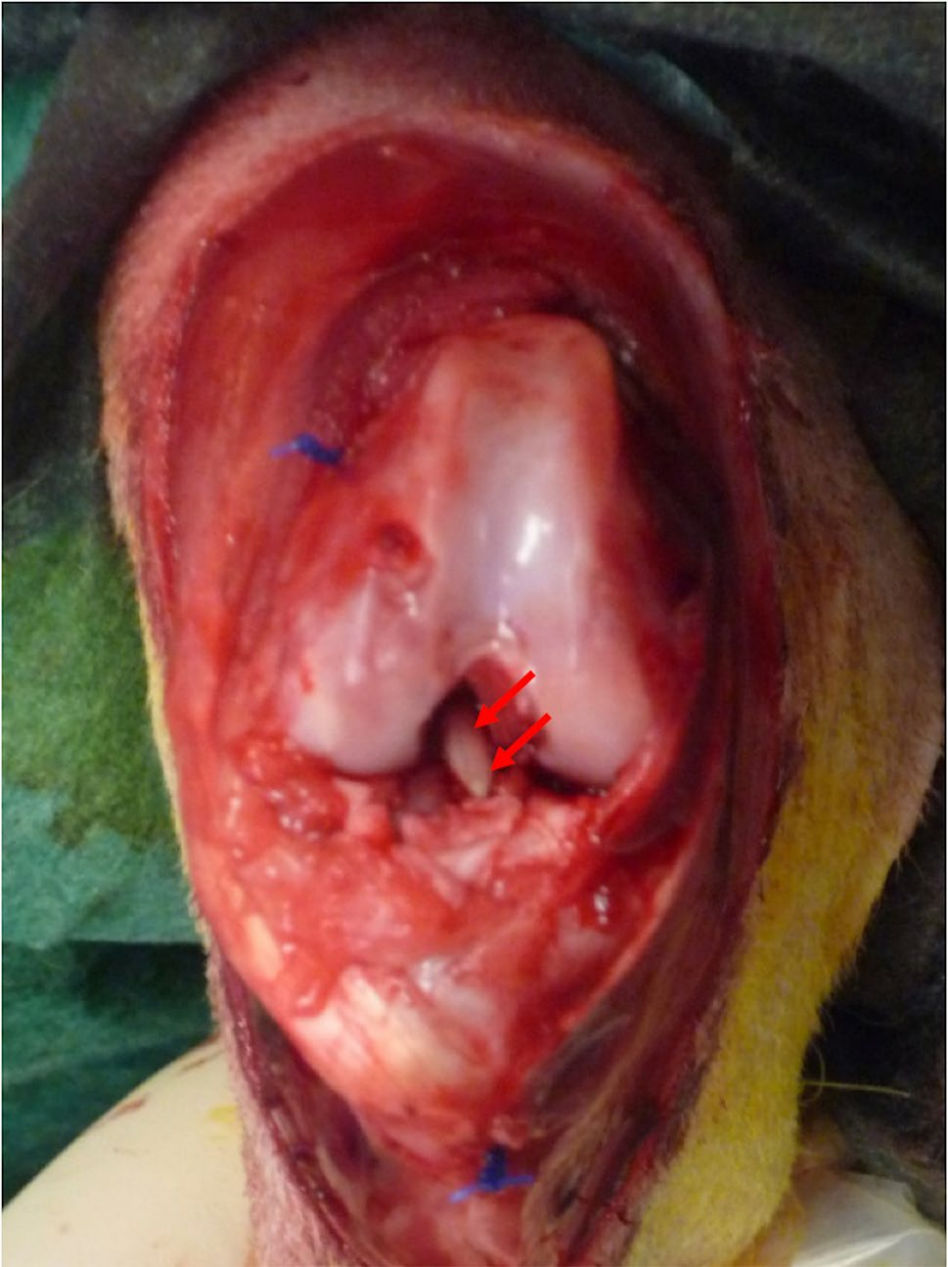
As a proof-of-concept study, *cond1* Sc-CO_2_-treated-EDL tendon was prepared as a replacement construct for the damaged ACL in a rabbit model. Macroscopic appearance 12 days after implantation in the right knee. Red arrows: ACL graft.

Because of the satisfactory results of the in vitro experimentation, Sc-CO_2_-treated tendons were implanted in vivo in order to: (1) test the feasibility of the technique; (2) assess the inflammatory reaction of the recipient animals; and (3) determine the gross appearance of the new ligament scaffold. Incorporation of the treated scaffolds into the rabbit joint was analyzed macroscopically 12 days post-implantation^52^. Dissection revealed a continuous scaffold graft with good macroscopical appearance: firm, glistening and under tension in the flexed joint. The joint fluid lacked an inflammatory appearance, with the synovium appearing healthy and the joint fluid clear and non-infected (Fig. 6).

This study does have limitations. This was mainly an in vitro study that did not investigate the mechanical behaviour of treated tendons after implantation. Sc-CO_2_ treated tendons were implanted in a rabbit model, but were only evaluated macroscopically. Also, we did not evaluate the immune response to the grafted Sc-CO_2_-treated tendons. In vitro, we only evaluated 2 experimental conditions (*cond1* and *cond2*); supercritical process parameters could be further optimized to improve the mechanical properties as compared to native tissues. Finally, the study was performed using rabbit tendons instead of human tissues. The procurement of human tendons for such a study would have been possible from cadavers, who are usually aged, and potentially have degenerative tendon tears^53^. By using animal tendons, we were able to standardize the procedure, and to implant treated scaffolds in the same species.

In summary, in view of the negative effects of irradiation on soft tissues, Sc-CO_2_ emerges as a powerful alternative and innovative technique to obtain tissue grafts pertinent ACL tissue engineering applications. In the present study, we have designed and produced a “smart” scaffold for ACL tissue reconstruction applications using Sc-CO_2_. This new material preserved the biomechanical properties, collagen crosslinking, collagen content, and histological structure of the native highly-tensile ligament tissue. Lastly, we have demonstrated biocompatibility and feasibility of the scaffold in vivo in a white rabbit ACL model. Additional large-scale animal studies may be needed to investigate the long-term integration, structural stability and biocompatibility of the novel grafts. However, the properties of the novel material make this product an innovative solution to clinical and scientific problems of ACL reconstruction.
